# Dense RGB-D SLAM with Multiple Cameras

**DOI:** 10.3390/s18072118

**Published:** 2018-07-02

**Authors:** Xinrui Meng, Wei Gao, Zhanyi Hu

**Affiliations:** 1 National Laboratory of Pattern Recognition (NLPR), Institute of Automation, Chinese Academy of Sciences, Beijing 100190, China; xinrui.meng@nlpr.ia.ac.cn (X.M.); huzy@nlpr.ia.ac.cn (Z.H.); 2 School of Artificial Intelligence, University of Chinese Academy of Sciences, Beijing 100049, China

**Keywords:** multi-camera, SLAM, calibration, RGB-D

## Abstract

A multi-camera dense RGB-D SLAM (simultaneous localization and mapping) system has the potential both to speed up scene reconstruction and to improve localization accuracy, thanks to multiple mounted sensors and an enlarged effective field of view. To effectively tap the potential of the system, two issues must be understood: first, how to calibrate the system where sensors usually shares small or no common field of view to maximally increase the effective field of view; second, how to fuse the location information from different sensors. In this work, a three-Kinect system is reported. For system calibration, two kinds of calibration methods are proposed, one is suitable for system with inertial measurement unit (IMU) using an improved hand–eye calibration method, the other for pure visual SLAM without any other auxiliary sensors. In the RGB-D SLAM stage, we extend and improve a state-of-art single RGB-D SLAM method to multi-camera system. We track the multiple cameras’ poses independently and select the one with the pose minimal-error as the reference pose at each moment to correct other cameras’ poses. To optimize the initial estimated pose, we improve the deformation graph by adding an attribute of device number to distinguish surfels built by different cameras and do deformations according to the device number. We verify the accuracy of our extrinsic calibration methods in the experiment section and show the satisfactory reconstructed models by our multi-camera dense RGB-D SLAM. The RMSE (root-mean-square error) of the lengths measured in our reconstructed mode is 1.55 cm (similar to the state-of-art single camera RGB-D SLAM systems).

## 1. Introduction

An important characteristic in visual RGB-D SLAM for accurate localization is a wide field of view, and a wide field of view system can not only improve the RGB-D camera pose tracking but also increase the efficiency of map building. Nowadays, RGB-D camera electronics get cheaper and smaller, using multiple RGB-D cameras in a robot or other platform is highly feasible, which provides potential for a wide field of view.

To achieve a wide field of view system by multiple RGB-D cameras, we need to do extrinsic calibration first between different RGB-D cameras. Villena-Martínez et al. [1] made a comparative study of different calibration methods. They focus on the intrinsic and extrinsic parameters between RGB sensor and depth senor. In our work, we suppose the above parameters have been calibrated, and focus on the extrinsic calibration between different RGB-D cameras. Most of the extrinsic calibration methods make use of the overlapping fields of view of the cameras. However, in robotic and unmanned fields, cameras are usually mounted around the rig in a ring and point outwards with little overlapping fields of view. It is not easy to calibrate such a system using existing calibration toolboxes by chessboard or circular-dot patterns [2] due to the minimal overlapping fields of view. Accordingly, Li et al. [3] designed a feature descriptor-based calibration pattern which is easy of detection even when the cameras observe partially of the board. Su et al. [4] proposed a novel algorithm to calibrate the RGB-D camera networks by using a spherical calibration object. Recently, as inertial sensors appear on robots, hand–eye calibration usually be used to calibrate the extrinsic parameters of the cameras. Tsai et al. [5] proposed a classical hand–eye calibration approach which requires the sensors rotate around at least two different axes. In [6,7,8], an improved hand–eye calibration algorithm was proposed where the sensors only rotate around one axis. In addition to hand–eye calibration, some visual method based on reconstruction can also calibrate non-overlapping multi-camera-rig. Esquivel et al. [9] performed individual structure from motion computation for each camera separately, then aligned the trajectories in 3D to estimate the relative camera poses. Carrera et al. [10] matched the trajectories by feature matching, estimated the initial transformation under random sample consensus (RANSAC) paradigm, and optimized the estimation using bundle adjustment. In our work, we use two different methods for calibrating non-overlapping fields of view RGB-D cameras. One is based on hand–eye calibration, which is for the system with inertial odometer. The other is based on visual RGB-D SLAM, using pose graph optimization to estimate the extrinsic parameters between RGB-D cameras without resorting to any other auxiliary.

Some methods of visual RGB-D SLAM use one RGB-D camera for visual odometry and mapping. Di et al. [11] proposed a new RGB-D SLAM based on extended bundle adjustment with integrated 2D and 3D information. Tang et al. [12] presented a novel approach to geometrically integrate the depth scene and RGB scene to enlarge the measurement distance of RGB-D sensors and enrich the details of model generated from depth images. Fu et al. [13] proposed a real-time dense mapping system using a local map and a global map with surfels. Huang et al. [14] described a system for visual odometry and mapping using an RGB-D camera, and its application to autonomous flight. Other methods used multiple cameras to improve the robustness of localization. Early traditional offline approaches use multi-camera system in structure from motion research [15]. Kaess et al. [16] presented a sparse SLAM approach, suitable for real-time reconstruction from multi-camera configurations. Sola et al. [17] proposed a multi-camera visual SLAM method using the extended kalman filter for simultaneous localization and mapping (EKF-SLAM). Urban et al. [18] extended the state-of-the-art oriented fast and rotated brief for simultaneous localization and mapping (ORB-SLAM) to a multi-fisheye camera system. However, the above methods all use multi-camera in sparse SLAM system, which can improve the localization accuracy but lack the efficiency for real-time 3D reconstruction. We propose a multi-camera dense RGB-D SLAM system, which can not only position the robot accurately but also build the dense 3D model in real time efficiently.

**Figure 1:**
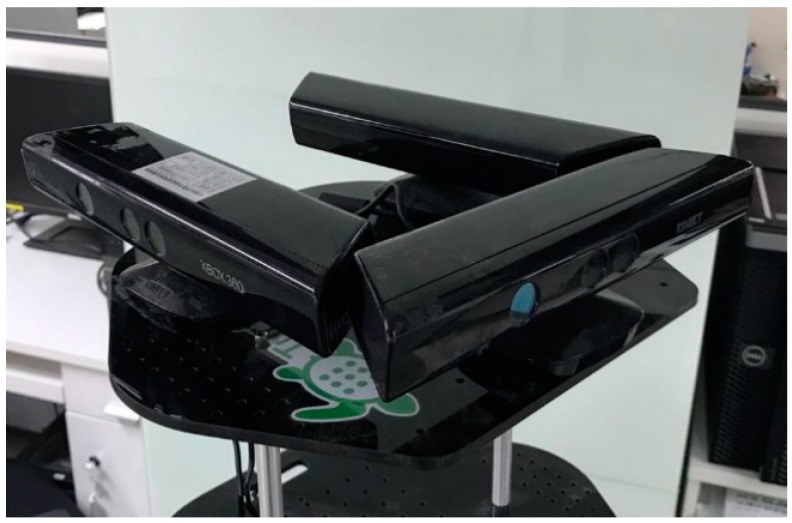
Example of three-Kinect arrangement.

In this work, we report a multi-camera dense RGB-D SLAM system. In our system, multiple RGB-D cameras are mounted around a rig in a ring as Figure 1. All cameras are linked to a workstation using cables. The multi-camera-rig can be fixed to a robot or a tripod with a pulley and capture the images in synchronization. Alexiadis et al. [19] proposed a method to more precisely synchronize the cameras using an audio synchronization scheme. Because the robot or tripod are moved slowly and all RGB-D cameras are linked the same computer, the images captured are considered to be synchronized and we do not adopt [19]. Next, we estimate the extrinsic parameters of the multi-camera-rig using two automatic procedures. One is for a robot with inertial sensors system, the other is for a tripod with the camera only system. Then we track each camera’s pose independently and transform them into a fixed coordinate system. We make the cameras’ poses assist each other by the known extrinsic parameters to enhance the robustness of camera pose tracking. In the pose optimization section, we use an improved deformation graph [20] to optimize the camera pose and align the map surface.

The main contributions of our work contain:

1. Two kinds of extrinsic calibration methods for three-Kinect system are proposed, one is suitable for system with IMU using an improved hand–eye calibration method, the other for pure visual SLAM without any other auxiliary sensors.
2. We extend the state-of-the-art ElasticFusion [20] to a multi-camera system to get a better dense RGB-D SLAM.

## 2. Extrinsic Calibration of Multiple Cameras

### 2.1. Odometer-Based Extrinsic Calibration

We run RGB-D visual odometry (VO) for each camera in a feature-rich scene to estimate a set of camera poses which is required for the subsequent step of hand–eye calibration. Our RGB-D VO method is similar to [21], which is the classical VO method for RGB-D SLAM. We perform a dense iterated close point (ICP) method to estimate the camera pose, using a projective data association algorithm [22] to obtain correspondence and a point-to-plane error metric for pose optimization. Then we solve the optimization problem based on the GPU’s parallelized processing pipeline. The point-to-plane error energy for the desired camera pose estimate **T** is

(1) $$E=\underset{u\in \Omega }{\sum }{\left(\left(Tv_{k}\left(u\right)-v_{k-1}\left(u\right)\right)\cdot n_{k-1}\right)}^{2}.$$

We track the current camera frame by aligning a live surface measurement ($v_{k}$, $n_{k}$) against the model prediction from the previous frame ($v_{k-1}$, $n_{k-1}$), where $\Omega \subset {\mathbb{N}}^{2}$ is the image space domain, **v** is vertex, **n** is normal, and *k* is the timestamp. With the VO method, we obtain a set of camera poses.

Then we use the hand–eye calibration method of [7] to estimate each camera-odometry transformation. The unknown camera-odometry transformation is estimated in two steps. In the first step, the rotation cost function is minimized to estimate the pitch and roll angles of the camera-odometry transformation. In the second step, the translation cost function is minimized to estimate the yaw angle and the camera-odometry translation. The relationship between camera and robot can be expressed as a rotation formula and a translation formula as

(2) qRiRi+1 qCR=qCR qCiCi+1,

(3) (R(qRiRi+1)−I) pCR=R(qCR) pCiCi+1−pRiRi+1.

In the above, the rotation is represented by quaternion, and the translation by a vector. The robot’s transformation between time *i* and time *i* + 1 is denoted by the vector pRiRi+1 and the unit quaternion qRiRi+1, which can be obtained from the robot’s inertial measurement unit. pCiCi+1 and qCiCi+1 represent the camera’s transformation between time *i* and time *i* + 1 which can be obtained by the above VO method. pCR and qCR represent the transformation between the robot and the camera. In the first step, we decompose the unknown unit quaternion qCR into three unit quaternions, corresponding to Z–X–Y. Euler angles *α*, *β*, *γ* as

(4) qCR=qz(α)qxy(β,γ).

Since both qRiRi+1 and $q_{z}\left(\alpha \right)$ represent rotations around the *z* axis, they satisfy commutative law. After simplifying Function (2), the rotation residual term becomes

(5) ηi=qRiRi+1 qxy(β,γ)−qxy(β,γ)qCiCi+1.

Then residual term $\eta_{i}$ is minimized to estimate $q_{xy}\left(\beta ,\gamma \right)$, similarly to [8]. In second step, we expand Function (3) and formulate a translation cost function to estimate the remaining unknowns

(6) εi=[cosϕi−1−sinϕisinϕicosϕi−1][pxpy]− [cosα−sinαsinαcosα][pi1pi2]+pRiRi+1,

where $\phi_{i}$ is the angle that the robot rotates around the *z* axis, $p_{x}$, $p_{y}$ are the translation components of the camera-odometry transformation, α is the yaw angle of the camera-odometry transformation, ${\left[\begin{array}{cc} p_{i1} & p_{i2} \end{array}\right]}^{T}$ denotes R(qxy(β,γ)) pCiCi+1. We minimize the residual term $\varepsilon_{i}$ by linear least squares method to estimate $p_{x}$, $p_{y}$, and α. Note that this method cannot estimate the translation component along the *z* axis.

We estimate the camera-odometry transformation for each camera using the above hand–eye calibration. Because all the cameras transform with respect to the same inertial measurement unit, we can estimate the extrinsic parameters between any two cameras via this inertial measurement unit

(7) $$H_{1-2}={H_{CR1}}^{-1}\;H_{CR2},$$

where $H_{CR1}$ and $H_{CR2}$ are the camera-odometry transformations of two cameras, $H_{1-2}$ is the extrinsic transformation matrix between these two cameras.

### 2.2. SLAM-Based Extrinsic Calibration

We run a simple multi-camera RGB-D slam to estimate the extrinsic parameters between the cameras by pose graph optimization. Firstly, we control the multi-camera rig rotate around itself to ensure different cameras have some overlapped view in the data capture stage. Then in the simple RGB-D visual odometry, we use SIFT features to match the images and solve the transformation T between two frames by classical ICP method [23] which use the following point-to-point error energy

(8) $$E=\sum_{i=1}^{N}{\left(p_{i}-Tq_{i}\right)}^{2},$$

where $\langle p_{i},q_{i}\rangle$ is a pair of matching points, *N* is the total number of matching points.

We divide the process of SLAM into two stages. In the front end stage, we run VO independently for each camera to estimate its initial pose. In the back end stage, we add a set of constraints for the camera pose and use pose graph optimization to adjust the pose.

**Figure 2:**
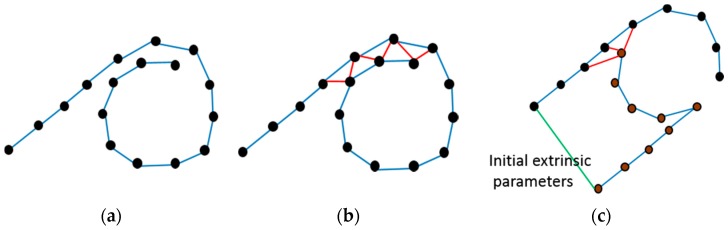
The sketch map of the pose graph. (**a**) is the initial pose graph obtained by VO where the blue edges are the transformations between two nearby keyframes. (**b**) is obtained after the closing loop detection and the red edges connect the frames satisfying the loop constraints. (**c**) denotes the relationship between two cameras, where black vertices are the poses of one camera and brown vertices belong to another camera, the green edge means the extrinsic parameters which can be initially set to the identity matrix.

During the VO process, some keyframes are chosen such that they have more inliers, and a new keyframe should be neither too far nor too close to the last chosen keyframe. The initial pose graph is obtained after VO, with the keyframes as vertices and the transformation between two neighboring keyframes as edges (Figure 2a). Then we add loop constraints to the graph. To find loop constraints, we determine all the keyframes before the current keyframe to see whether these keyframes have a successful feature matching with the current keyframe. If feature matching is successful, we add an edge between the two frames in the pose graph (Figure 2b). From the multiple cameras we choose one camera as the reference camera whose first frame’s coordinate system is defined as the world coordinate system. Then we add edges between the reference camera’s first pose and other cameras’ first poses separately, set the transformation matrix as the identity matrix (Figure 2c). We fix the reference camera’s first pose and optimize the pose graph by G2O (an open-source C++ framework for optimizing graph-based nonlinear error functions). After the optimization, we obtain all cameras’ first poses, which are the relative transformations between cameras and the reference camera. The extrinsic parameters of multiple cameras are estimated.

In the above procedure, if have initial extrinsic parameters—for example obtained from odometer-based calibration—we can set the edge value between the reference camera’s first pose and other cameras’ first poses with the available initial extrinsic parameters. By this way, the two extrinsic calibration methods are combined.

## 3. Multi-Camera RGB-D SLAM

### 3.1. Tracking

We extend the state-of-the-art ElasticFusion [20] to multi-camera system to get a better dense RGB-D SLAM. Before the visual odometry, we give each camera’s first frame an initial pose from the extrinsic calibration. Then we track the pose of each camera respectively by minimizing the geometric and photometric joint cost function

(9) $$E_{track}=E_{icp}+w_{rgb}E_{rgb},$$

where $w_{rgb}$ is the weight and was set empirically to 0.1 to reflect the difference in metrics used for ICP and RGB costs (meters and 8-bit intensity respectively). $E_{icp}$ is the cost over the point-to-plane error between 3D back-projected vertices

(10) $$E_{icp}=\underset{k}{\sum }{\left(\left(v^{k}-Tv_{t}^{k}\right)\cdot n^{k}\right)}^{2}.$$

$E_{rgb}$ is the cost over the photometric error between pixels

(11) $$E_{rgb}=\underset{u\in \Omega }{\sum }{\left(I\left(u,C_{t}\right)-I\left(\pi \left(K\;Tp\left(u,D_{t}\right)\right),C_{t-1}\right)\right)}^{2},$$

where $v_{t}^{k}$ is the back-projection of the *k*-th vertex in the current depth image, $v^{k}$ and $n^{k}$ are the corresponding vertex and normal represented in the last predicted depth image. Vertices are associated using projective data association. $C_{t}$ is the current color image and $C_{t-1}$ is the last predicted color image. I(u,C) refers the intensity value of a pixel **u** given a color image C. p(u,D) means the 3D back-projection of a point **u** given a depth map D. π(p) means the perspective projection of a 3D point **p**. We minimize the joint cost function $E_{track}$, obtain the transformation matrix **T**, and finally estimate the current pose of each camera.

In the multi-camera system, we can obtain multiple poses from different cameras at the same moment. We choose one pose with minimum joint error as the reference pose and compute other cameras’ poses from it. For example, if we have three cameras to capture images and have estimated their extrinsic parameters $H_{1-2}$, $H_{1-3}$ by extrinsic calibration method. At the current moment, the pose of camera 2 is the reference pose $P_{2}$, then other cameras’ poses are

(12) $$P_{1}=P_{2}\;{H_{1-2}}^{-1},$$

(13) $$P_{3}=P_{2}\;{H_{1-2}}^{-1}\;H_{1-3}.$$

Using the above undertaking, when one of the cameras track fails, the poses of other cameras can be used to prevent this pose from drifting. Actually, if only one camera is successful in tracking, all the other cameras’ poses can be computed.

### 3.2. Mapping

In the back end optimization part, we use the deformation graph to optimize the camera pose. The deformation graph is a non-rigid space map deformation method which is better than the pose graph optimization in the dense SLAM system. We extend the deformation graph method to a multi-camera system.

Similar to Elasticfusion [20], we represent the 3D model by an unordered list of surfels ℳ which possess some attributes, including position, normal, color, weight, radius, initial timestamp, last updated timestamp, and device number (which represent the first camera number to construct this surfel). The deformation graph is composed of nodes and edges. Nodes denoted as G, are randomly selected from surfels and each node has a position, a timestamp, a set of neighbor nodes, a device number, and an affine transformation (including a rotation matrix and a translation vector). The neighbors of each node make up the edges of the graph. After optimizing the deformation graph by the closed loops in the SLAM process [20], we use these nodes to deform the other surfels in the map. Because there are multiple cameras in the system, when a closed loop is detected, we need to confirm which camera’s frame trigger the closed loop and in the next step we only deform the surfels whose device numbers belong to the triggering camera.

${ℳ}_{d}$ is the surfel which has the same device number of d with the loop trigger frame. We want to deform it by some optimized deformation nodes. Firstly, in the set of deformation nodes with device number of d, we select a set of closest nodes to ${ℳ}_{d}$ in time and form a set. Then we choose some closest nodes in distance with ${ℳ}_{d}$ from the above set, and make up a new set of influencing nodes of surfel ${ℳ}_{d}$ as $ℐ\left({ℳ}_{d},G_{d}\right)$. We deform the position and normal of the surfel ${ℳ}_{d}$ by the above influencing nodes

(14) $$\hat{{ℳ}_{d}^{p}}=\sum_{n\in ℐ\left({ℳ}_{d},G_{d}\right)}\omega^{n}\left({ℳ}_{d}\right)\;\left[G_{d,n}^{R}\;\left({ℳ}_{d}^{p}-G_{d,n}^{g}\right)+G_{d,n}^{g}+G_{d,n}^{t}\right],$$

(15) $$\hat{{ℳ}_{d}^{nor}}=\sum_{n\in ℐ\left({ℳ}_{d},G_{d}\right)}\omega^{n}\left({ℳ}_{d}\right)\;{G_{d,n-1}^{R}}^{T}\;{ℳ}_{d}^{nor},$$

(16) $$\omega^{n}\left({ℳ}_{d}\right)={\left(1-\|{ℳ}_{d}^{p}-G_{d,n}^{g}{\|}_{2}/d_{max}\right)}^{2},$$

where ${ℳ}_{d}^{p}$ represents the surfel’s position before deformation, $\hat{{ℳ}_{d}^{p}}$ represents the surfel’s position after deformation; ${ℳ}_{d}^{nor}$ represents the surfel’s normal before deformation, $\hat{{ℳ}_{d}^{nor}}$ represents the surfel’s normal after deformation. $G_{d,n}$ denotes the n-th influencing node, $\omega^{n}\left({ℳ}_{d}\right)$ is the influence scalar of the node $G_{d,n}$, $G_{d,n}^{R}$ represents the node’s rotation matrix, $G_{d,n}^{g}$ represents the node’s position, $G_{d,n}^{t}$ represents the node’s translation vector. $d_{max}$ is the maximal distance from influence nodes to ${ℳ}_{d}$.

## 4. Experiment

**Figure 3:**
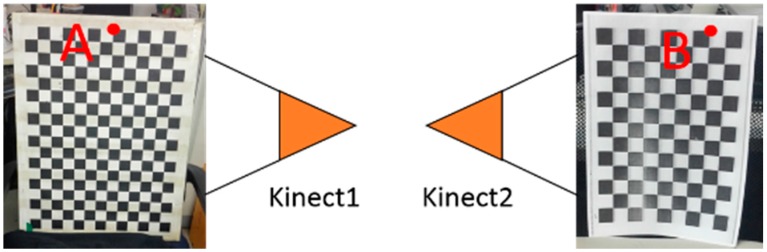
The placement of the two calibration boards in the two-Kinect extrinsic calibration accuracy experiment. A and B are the two corner points to be measured distance.

Firstly, we verify the accuracy of extrinsic calibration by a two-Kinect system, one Kinect towards the left and another towards the right, with approximate 180° relative rotation. In the calibration scene, we put the calibration board in each Kinect’s field of view respectively, which is shown in Figure 3. We measure the distance of the two boards from point A to point B (in Figure 3) by laser rangefinder and make it as the ground truth. Then we estimate the extrinsic parameters of the two Kinects by odometer-based and SLAM-based methods, separately and in combination, and build three dense 3D models using the above extrinsic parameters as cameras’ initial poses and measure the distance of the calibration boards in the three 3D models respectively. Table 1 compares the distance results generated by the three methods with ground truth. As shown in Table 1, the combination method of odometer and SLAM performs better.

For all experiments, we run our system on a desktop with an Intel Xeon E5-1620 CPU (DELL, Xiamen, China) at 3.7 GHZ, 32 GB of RAM, and an NVidia GeForce GTX 1060 GPU (ASUS, Suzhou, China) with 6 GB of memory.

**Figure 4:**
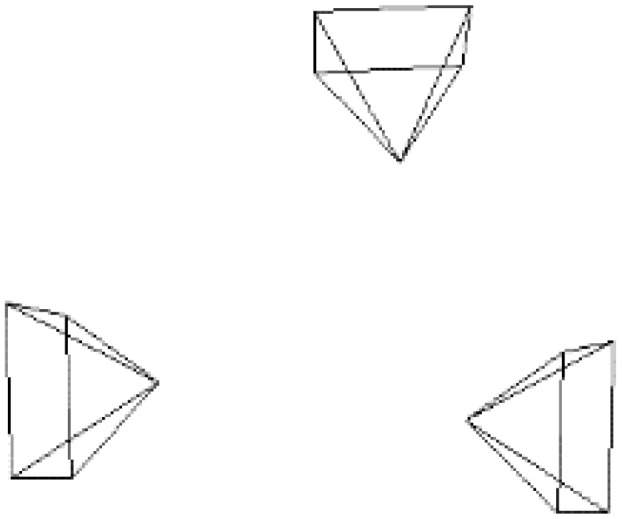
Relative positions of the three Kinects.

**Figure 5:**
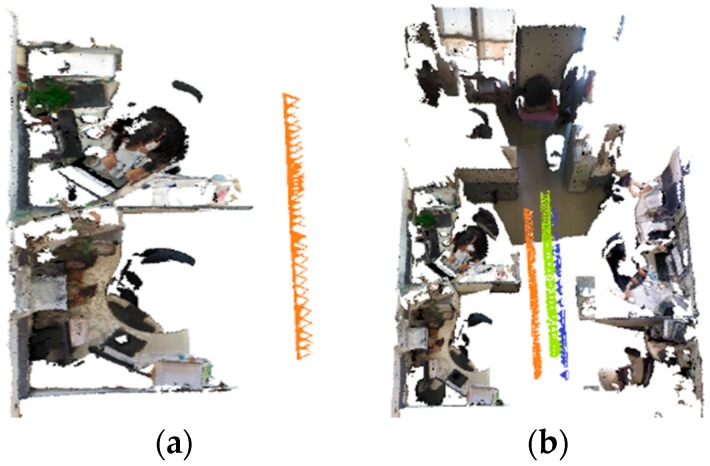
Comparison of single-camera SLAM result and three-camera SLAM result. (**a**) is a single-camera SLAM result, the movement trajectory is in orange. (**b**) is a three-camera SLAM result with the same movement trajectory as (**a**), different colors mean different camera trajectories.

**Figure 6:**
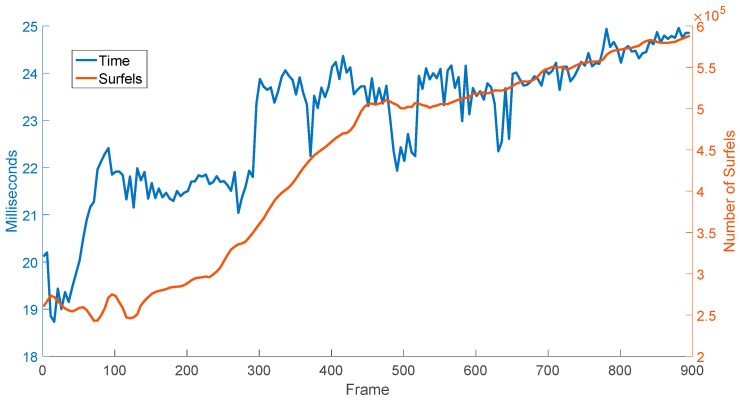
Frame time vs. number of surfels.

We use three Kinects to test the efficiency of the multi-camera RGB-D SLAM system. The three Kinects’ position relationships from the top view are shown in Figure 4. In the experiment, we compare the reconstructed result of single camera SLAM and three-camera SLAM with same movement of the rig. As shown in Figure 5, three-camera SLAM can build a larger map. Thus using multi-camera in RGB-D SLAM can improve the efficiency of reconstruction significantly. As shown in Figure 6, the execution time of our system increases with the number of surfels in the map, the overall average time is 23 ms per frame.

**Figure 7:**
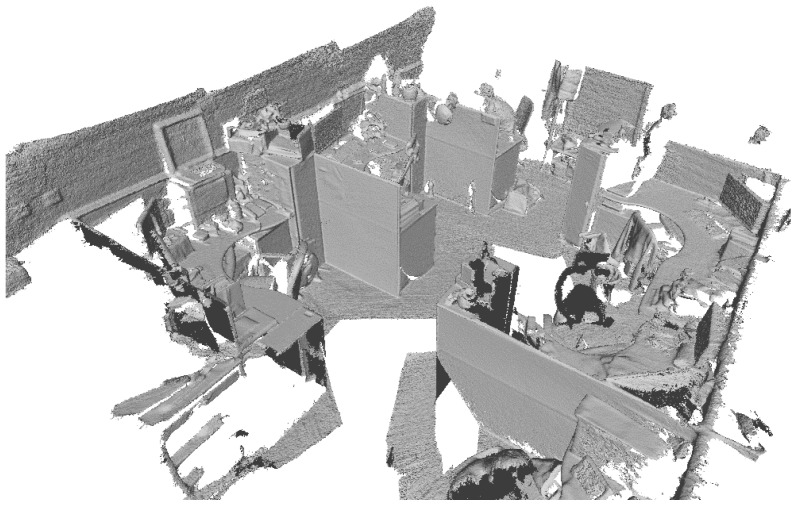
3D reconstruction result by InfiniTAM.

**Figure 8:**
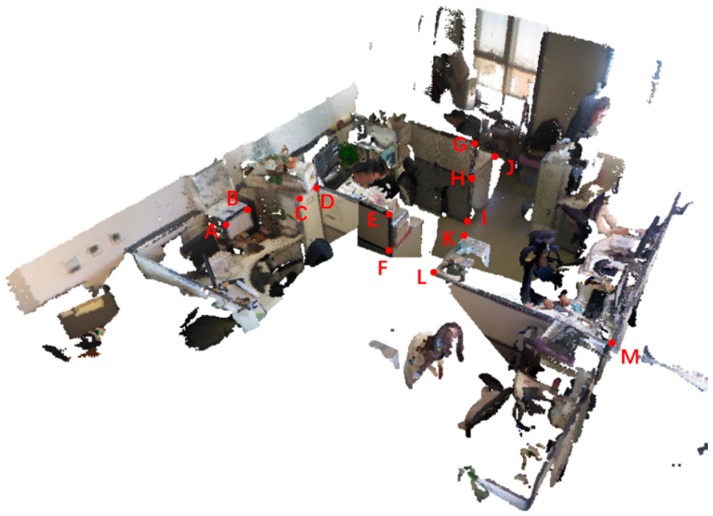
3D reconstruction result by our method and the measured points. A–M are the end points of line segments.

We also use monocular RGB-D SLAM InfiniTAM [24] to reconstruct the 3D model (Figure 7) of the same area as Figure 5. To illustrate the reconstruction accuracy of real-world scenes, we compare the actual lengths of seven line segments on the scenes with the lengths measured in the reconstructed model. The selected line segments and their lengths are depicted in Figure 8 and Table 2. The RMSE of the lengths measured in our reconstructed mode is 1.55 cm and the RMSE of the lengths measured in the reconstructed mode by InfiniTAM is 1.34 cm. From which we know that our reconstruction accuracy is fairly accurate.

**Figure 9:**
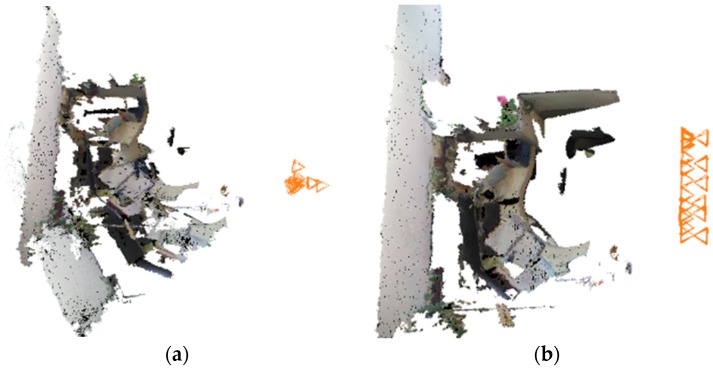
Comparison of single-camera SLAM result and two-camera SLAM result when one of the cameras is occluded. (**a**) is the single-camera SLAM result; (**b**) is the two-camera SLAM result.

To verify the robustness of our multi-camera RGB-D SLAM system, we make a pedestrian occlude one of the cameras during the SLAM process and compare the result of single-camera SLAM and two-camera SLAM. As shown in Figure 9, the single-camera SLAM fails to track the camera pose as a pedestrian occludes the camera and the two-camera SLAM can continue tracking with the help of the other camera.

From the above experiments, we firstly demonstrate the accurate extrinsic calibration with multiple cameras which share no common field of view for two different applications, one is for a robot with inertial sensors system, the other is for a tripod with the camera only system. The accuracy of odometer-based calibration method is more accurate than SLAM-based calibration method. However, the accuracy of combined method is the most accurate method. How to choose the calibration method is based on the hardware. Secondly, we show that our tracking and mapping method can make an accurate 3D reconstruction and the RMSE of the lengths measured in our reconstructed mode is 1.55 cm (similar to the state-of-art single camera RGB-D SLAM systems). The overall average processing time is 23 ms per frame and can be used for real-time operation. Thirdly, we make the pipeline robust to breaks in monocular visual odometry which occur in areas with low texture or occluded by pedestrians.

## 5. Conclusions

In this paper, we propose a multi-camera dense RGB-D SLAM system. We use two kinds of extrinsic calibrations, one for system with inertial measurement unit, and the other for system with only camera sensors. These two kinds of calibration methods both can estimate the extrinsic parameters between cameras lacking a common field of view. After calibration, we extend a state-of-the-art dense single RGB-D SLAM method [20] to multi-camera system. In the tracking stage, multiple cameras do visual odometry independently and the minimal-error camera pose is chosen as the reference pose and used to correct the other cameras’ poses in case of some camera tracking fails. In the mapping stage, we add a device number attribute to the map surfels, and surfels with different device numbers do different deformations. It is shown our multi-camera dense RGB-D SLAM greatly increased the efficiency of reconstruction as well as improve the accuracy and robustness of localization. In the future, we will consider a multiple-sensor-fusion approach to improve the robustness of the system.

## Figures and Tables

**Table 1:** Accuracy comparison among three extrinsic calibration methods

Sequence	Ground Truth	Odometer Calib	SLAM Calib	Odo + SLAM Calib
1	2.510 m	2.489 m	2.481 m	2.490 m
2	1.969 m	1.953 m	1.940 m	1.955 m

**Table 2:** Comparison between the actual lengths of seven line segments with the lengths measured in the reconstructed model

Line Segment	Length in Our Reconstructed Model	Length in the Reconstructed Model by InfiniTAM	Actual Length
AB	28.64 cm	27.51 cm	29.50 cm
CD	26.37 cm	27.02 cm	27.00 cm
EF	44.80 cm	44.06 cm	44.30 cm
GI	121.13 cm	118.48 cm	118.50 cm
HJ	62.84 cm	61.82 cm	62.10 cm
KL	63.44 cm	61.20 cm	62.30 cm
LM	168.37 cm	172.87 cm	170.50 cm
RMSE	1.55 cm	1.34 cm	/
